# Comprehensive analysis of N6-methyladenosine-related RNA methylation in the mouse hippocampus after acquired hearing loss

**DOI:** 10.1186/s12864-023-09697-4

**Published:** 2023-09-27

**Authors:** Xuehua Zhou, Lin Jin, Yufeng Li, Yiru Wang, Wen Li, Xia Shen

**Affiliations:** 1 Department of Anesthesiology, Eye & ENT Hospital, Fudan University, 83 Fenyang Road, 200031 Shanghai, China; 2 ENT Institute, Department of Otorhinolaryngology, Eye & ENT Hospital, Fudan University, 83 Fenyang Road, 200031 Shanghai, China

**Keywords:** Hearing loss, Cognition impairment, Hippocampus, m6A methylation, MeRIP-Seq, RNA-Seq

## Abstract

**Background:**

The mechanism underlying cognitive impairment after hearing loss (HL) remains unclear. N6-methyladenosine (m6A) is involved in many neurodegenerative diseases; however, its role in cognitive impairment after HL has not yet been investigated. Therefore, we aimed to analyze the m6A modification profile of the mouse hippocampus after HL exposure. A mouse model of neomycin-induced HL was established. An auditory brainstem-response test was utilized for detecting hearing threshold. The passive avoidance test was served as the mean for evaluating cognitive function. The m6A-regulated enzyme expression levels were analyzed by using reverse transcription quantitative real-time polymerase chain reaction and western blot analyses. RNA sequencing (RNA-Seq) and methylated RNA immunoprecipitation sequencing (MeRIP-Seq) were performed with the aim of investigating gene expression differences and m6A modification in the mouse hippocampus.

**Results:**

Neomycin administration induced severe HL in mice. At four months of age, the mice in the HL group showed poorer cognitive performance than the mice in the control group. *METTL14*, *WTAP*, and *YTHDF2* mRNA levels were downregulated in the hippocampi of HL mice, whereas *ALKBH5 and FTO* mRNA levels were significantly upregulated. At the protein level, METTL3 and FTO were significantly upregulated. Methylated RNA immunoprecipitation sequencing analysis revealed 387 and 361 m6A hypermethylation and hypomethylation peaks, respectively. Moreover, combined analysis of mRNA expression levels and m6A peaks revealed eight mRNAs with significantly changed expression levels and methylation.

**Conclusions:**

Our findings revealed the m6A transcriptome-wide profile in the hippocampus of HL mice, which may provide a basis for understanding the association between HL and cognitive impairment from the perspective of epigenetic modifications.

**Supplementary Information:**

The online version contains supplementary material available at 10.1186/s12864-023-09697-4.

## Background

As the most common sensory deficit, hearing loss (HL) affects more than one billion people worldwide and has become the third leading cause of disability [1]. In children, HL can hinder daily communications, lower the quality of life, potentially delay spoken language development, and lead to cognitive impairment later in life [2]. Children with mild HL show impaired learning and memory function [3], supporting the link between HL and cognitive impairment. In rodent models of HL, pathological changes indicative of cognitive impairment (e.g., inhibited neurogenesis, increased tau protein phosphorylation, and elevated neuroinflammation) have been found in the hippocampus [4, 5]. Despite the abundance of evidence supporting a link between HL and cognitive dysfunction, however, the specific molecular mechanism still remains unsolved.

Most recently, RNA methylation-mediated RNA posttranscriptional modification has attracted much attention as an important epigenetic modification [6]. N6-methyladenosine (m6A) methylation, the most frequent modification of eukaryotic RNA, has considered to be an important epigenetic modification and plays essential roles in regulating the location, stability, splicing, translation, transport of mRNA [7, 8]. In the brains of mammals, m6A methylation can be regulated reversibly and dynamically by demethylases (erasers) and methyltransferases (writers), where m6A methylation is recognized by other proteins (readers) [9]. Erasers (α-ketoglutarate-dependent dioxygenase alkB homolog 5 [ALKBH5] and fat-mass and obesity-associated protein [FTO]) catalyze the removal of m6A, writers (methyltransferase-like 14 [METTL14], methyltransferase-like 3 [METTL3], and Wilms tumor 1-associating protein [WTAP]) catalyze the addition of m6A, and readers (YTH domain family, like YTHDF1, YTHDF2, and YTHDF3) respond or recognize m6A specifically [10–13].

An increasing number of studies highlighted the critical significance of m6A modification in the development al nervous system, and its dysregulation has been shown to be related to neurodegenerative and neurodevelopmental diseases [14, 15]. m6A participates in neurobiological processes, such as neurogenesis, neurodevelopment, synaptic plasticity, learning, and memory [16–19]. Furthermore, various m6A players have been found to be mutated or dysregulated both in neurological degeneration disease, such as AD, HD, and depression [20–22]. In the mammalian central nervous system, stimulus-dependent m6A regulation occurs in response to learning process, injury, and sensory experience [14, 23], indicating that m6A modifications may be critical for the pathogenesis of cognitive impairment after sensory deprivation, such as HL. However, the involvement of m6A methylation in cognitive impairment after HL has not yet been investigated.

In this experiment, we sought to build up a mouse model of neomycin-induced HL and perform RNA sequencing (RNA-seq) and methylated RNA immunoprecipitation sequencing (MeRIP-seq) to observe mRNA expression patterns and transcriptome-wide m6A changes in hippocampi of HL and control (Con) mice. To predict the functions of differentially expressed RNAs, Gene Ontology (GO) and Kyoto Encyclopedia of Genes and Genomes (KEGG) pathway analyses were conducted. This study might provide insights on m6A-modified transcripts that are key for the development of cognitive impairment after HL.

## Results

### Establishing a mouse model of neomycin-induced HL

**Fig. 1:**
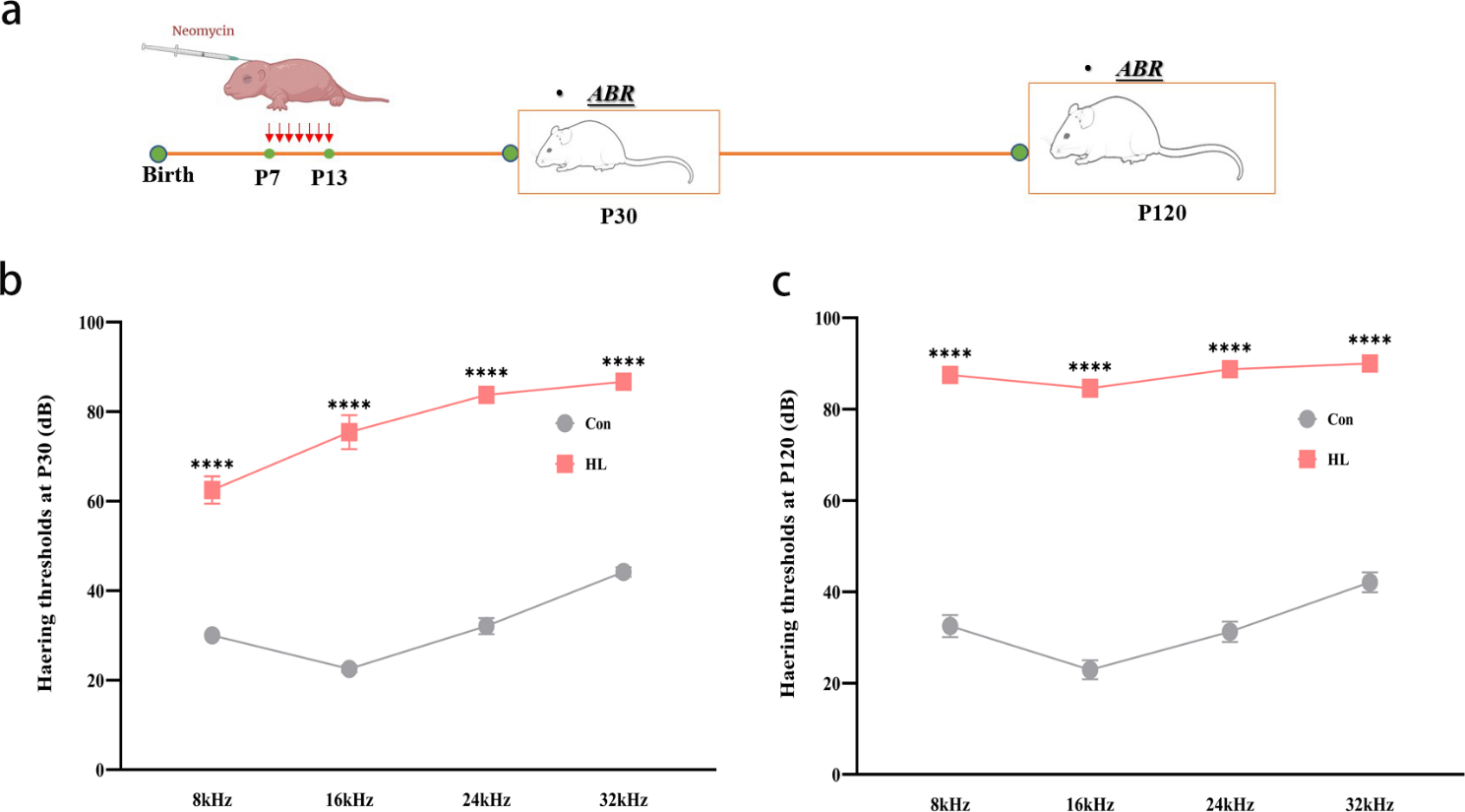
Successful establishment of a mouse model of neomycin-induced hearing loss (HL). **(a)** Time points for administering neomycin and performing the ABR test. **(b)** ABR thresholds of P30 mice. At P30, mice in the HL group had lower ABR thresholds than those in the Con group at 8, 16, 24, and 32 kHz. **(c)** ABR thresholds of P120 mice. At P120, mice in the HL group had lower ABR thresholds than those in the Con group at 8, 16, 24, and 32 kHz. The data shown were analyzed using an unpaired t-test and represent the mean ± SEM. ****P < 0.0001. n = 9 mice per group. Con, control; HL, hearing loss. ABR, auditory brainstem response; kHz, 1000 Hz; dB, decibel

To examine whether neomycin induces HL in mice, we performed auditory brainstem response (ABR) testing at postnatal day (P) 30 and P120 (Fig. 1a). At P30, HL mice had higher ABR thresholds than the Con group at different frequencies: 8 kHz (62.50 ± 3.05 vs. 30.00 ± 0.87 dB, *P* < 0.0001), 16 kHz (75.42 ± 3.82 vs. 22.50 ± 2.61 dB, *P* < 0.0001), 24 kHz (83.75 ± 1.52 vs. 32.08 ± 1.79 dB, *P* < 0.0001), and 32 kHz (86.67 ± 3.89 vs. 44.17 ± 1.04 dB, *P* < 0.0001), as shown in Fig. 1b. At P120, the ABR thresholds were also elevated at different frequencies in the experimental group: 8 kHz (87.5 ± 1.44 vs. 32.50 ± 2.42 dB, *P* < 0.0001), 16 kHz (84.58 ± 1.89 vs. 22.92 ± 2.08 dB, *P* < 0.0001), 24 kHz (88.75 ± 0.65 vs. 31.25 ± 2.23 dB, *P* < 0.0001), and 32 kHz (90 ± 0 vs. 42.08 ± 2.17 dB, *P* < 0.0001) (Fig. 1c). These findings indicate that neomycin successfully induced severe and irreversible HL in mice.

### Occurrence of cognitive impairment after HL

**Fig. 2:**
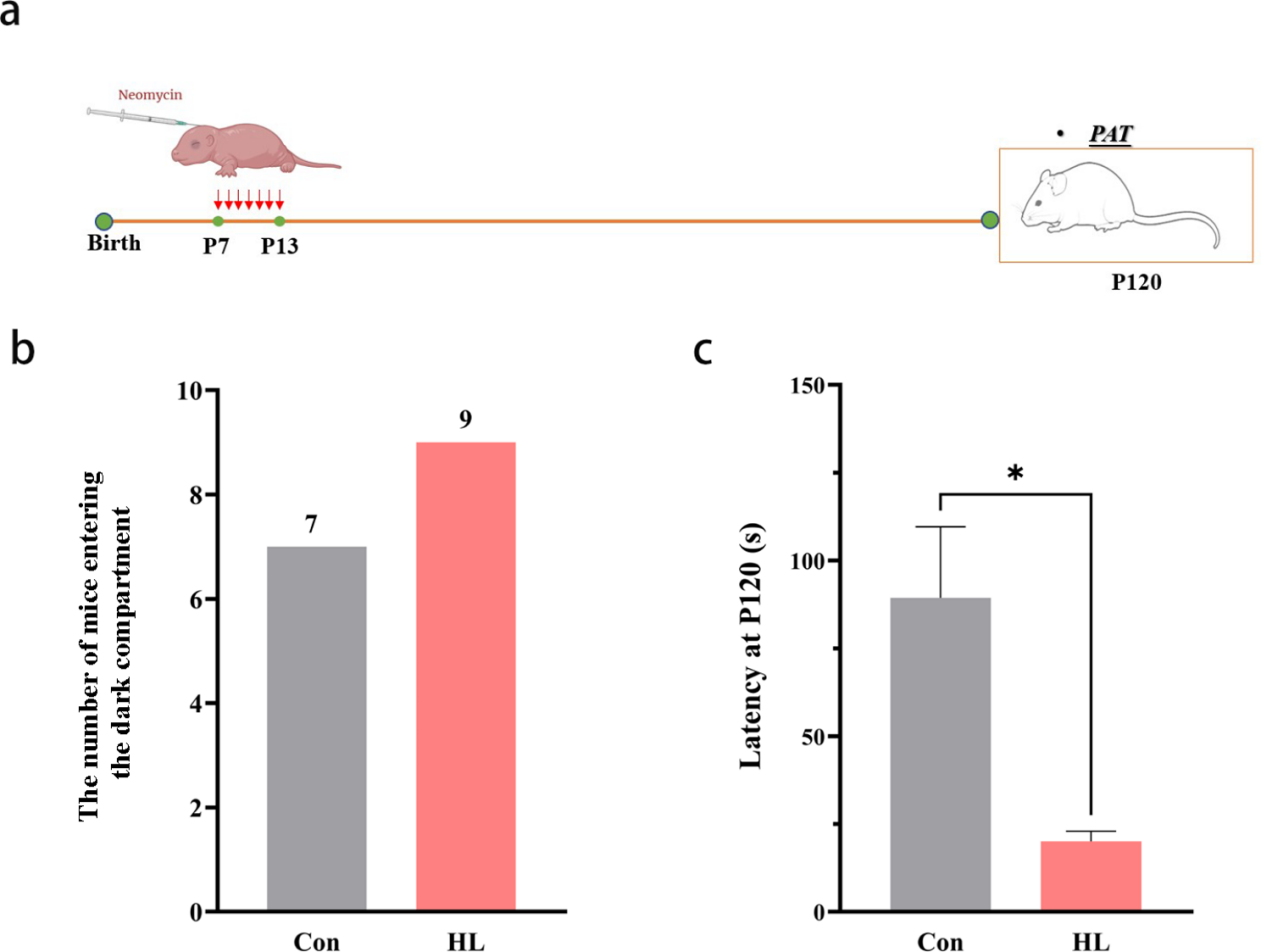
Occurrence of cognitive impairment after HL. **(a)** Time points for administering neomycin and conducting the PAT. **(b)** At P120, nine and seven mice in the HL and Con groups entered the dark room, respectively. **(c)** Latency of P120 mice. The latency of the HL group was significantly lower than that of the Con group. The data shown were analyzed using an unpaired t-test and represent the mean ± SEM. *P < 0.05. n = 9 mice per group. Con, control; HL, hearing loss

We conducted a passive avoidance test (PAT) in order to assess the cognitive function of mice at P120 (Fig. 2a). During the training period, the latencies of both groups were comparable. During the test period, seven mice (77.8%) in the Con group got into the dark compartment within the assigned time (300 s), while nine mice (100%) in the HL group (Fig. 2b). Thus, the HL mice had shorter latency (*P* < 0.05; Fig. 2c). These results mean that cognitive impairment occurred after HL.

### Overview of m6A RNA methylation in mouse hippocampi after HL

MeRIP-seq was performed to investigate the transcriptome-wide m6A-seq analysis. An average of 8.47 Gb sequencing data of the input samples along with 7.86 Gb sequencing data in the MeRIP-seq were obtained (Additional file 1). After discarding low-quality reads and bases, adaptor sequences, and poly-N sequences, the input and the MeRIP-seq samples contained (on average) 6.44 and 6.97 Gb of sequencing data, respectively (Additional file 1). The majority (95.03%) of the left high-quality reads in each sample could be mapped to the reference mouse genome (GRCm39). Clean reads that had unique alignments to the mouse genome (89.41%) were reserved for further analyses, and clean reads with multiple alignments (5.89%) were eliminated (Additional file 2).

**Fig. 3:**
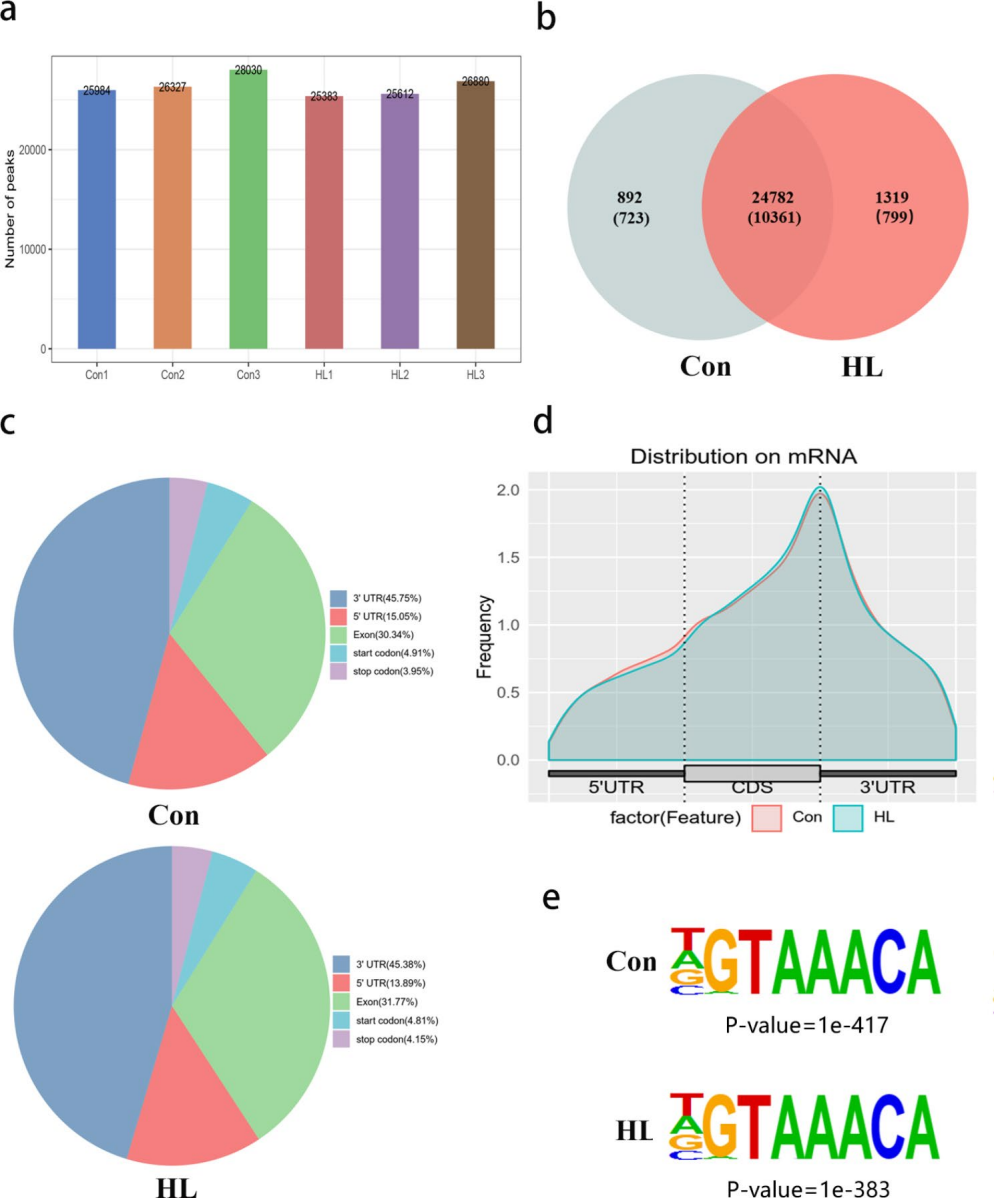
Overview of m6A RNA methylation in mouse hippocampi after HL. **(a)** The number of peaks in each sample in both groups. An average of 26,780 and 25,958 peaks were detected in the Con and HL group, respectively. **(b)** Overlapping mRNA m6A peaks between the Con and HL groups are displayed with a Venn diagram. **(c)** The distribution of m6A peaks in the transcripts is displayed with a density curve for the 5′-UTR, CDS, and 3′-UTR. **(d)** The ratios of m6A-peak distributions in the Con and HL samples are displayed in pie charts. **(e)** Detection of a common motif enriched across the m6A peaks in both groups

MeRIP-seq analysis revealed that the Con and HL group had an average of 26,780 and 25,958 peaks, respectively (Fig. 3a). We identified 25,674 MeRIP regions enriched in m6A peaks, and these peaks were identified within 11,084 mRNA transcripts of coding genes. In addition, 26,101 m6A peaks within 11,160 mRNAs were detected in HL group. Among the genes with m6A peaks, 10,361 mRNAs overlapped between both groups (Fig. 3b), showing that m6A methylation was abundant in the hippocampus. The distribution patterns regarding m6A methylation peaks within mRNAs were analyzed for the entire transcriptome. In both the Con and HL groups, we observed that the m6A peaks were predominantly located within the coding sequences (CDS), 5ʹ-untranslated region (UTR), and 3ʹ-UTR of the mRNA molecules (Fig. 3c, d). HOMER software was applied to observe if the m6A peaks owned the classic “RRACH” motif (R: G/A, H: U/ A/ C). In this research, the representative “AAACA” motif was enriched among the m6A peaks detected in both groups (Fig. 3e).

### Altered m6A modifications in mouse hippocampi after HL

**Fig. 4:**
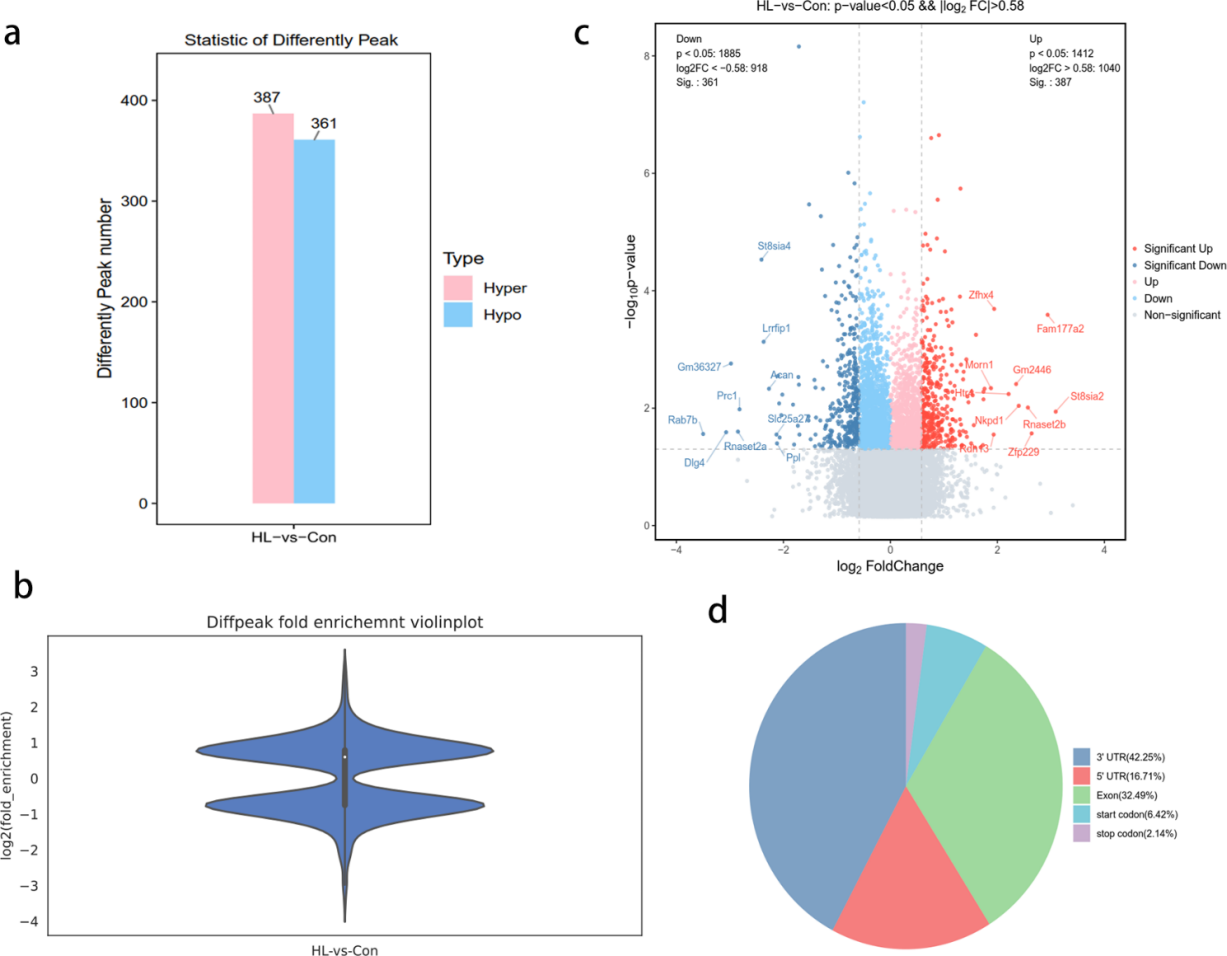
The distribution of peaks reflecting differential m6A methylation in mouse hippocampi after HL. **(a)** The number of peaks related to differential m6A methylation after HL. We detected 387 and 361 peaks reflecting m6A hypermethylation and hypomethylation, respectively (*P* < 0.05, |log_2_ FC| > 0.58) in mouse hippocampi after HL. **(b)** Violin plot displaying the fold-enrichments of peaks reflecting differential m6A methylation. **(c)** Volcano plot displaying peaks reflecting differential m6A methylation (*P* < 0.05, |log_2_ FC| > 0.58) in mouse hippocampi after HL. Red plots, peaks reflecting m6A hypermethylation; blue plots, peaks reflecting m6A hypomethylation. **(d)** Pie charts displaying the ratios of peaks reflecting differential m6A methylation and m6A peak distributions are displayed by pie charts. Diffpeak, differentially methylated m6A peak; FC, fold-change

We identified 748 peaks that showed differential m6A methylation between the HL and Con groups (|log_2_ fold change (FC)| > 0.58 and *P* < 0.05; Additional file 3). Among them, 387 m6A peaks were significantly hypermethylated, while 361 were significantly hypomethylated in the HL group (Fig. 4a). Table 1 presents the top 20 genes with hypermethylated and hypomethylated m6A methylation region. Furthermore, significantly hypomethylated and hypermethylated mRNAs are depicted by the violin plot and volcano plot (Fig. 4b, c). The distribution patterns of m6A peaks were classified into five distinct types of transcript parts: the 3ʹ-UTR, CDS, stop codon segment, start codon segment, and 5ʹ-UTR. Our findings indicated that those m6A sites were typically located in the 3ʹ-UTR and CDS regions (Fig. 4d).

**Table 1:** The representative changed m6A methylation peak. (Top 20)

Gene Id	Chromsome	P value	Log2fold change	Start	End	Width	Regulation
*St8sia2*	7	0.011481536	3.09	73,592,844	73,592,994	150	UP
*Fam177a2*	12	0.00025704	2.94	55,263,655	55,263,854	199	UP
*Zfp229*	17	0.026915348	2.64	21,966,955	21,967,105	150	UP
*Rnaset2b*	17	0.009772372	2.57	7,259,127	7,265,303	194	UP
*Nkpd1*	7	0.009120108	2.4	19,258,485	19,258,635	150	UP
*Gm2446*	12	0.003890451	2.35	55,263,649	55,264,059	196	UP
*Htr4*	18	0.005754399	2.21	62,635,349	62,635,499	150	UP
*Zfhx4*	3	0.000204174	1.94	5,467,216	5,467,416	200	UP
*Rdh13*	7	0.028183829	1.93	4,449,241	4,449,392	151	UP
*Morn1*	4	0.004570882	1.88	155,184,691	155,184,842	151	UP
*Rab7b*	1	0.027542287	-3.5	131,640,859	131,641,010	151	Down
*Dlg4*	11	0.025703958	-3.07	69,919,726	69,919,927	201	Down
*Gm36327*	6	0.001737801	-2.98	47,655,432	47,655,582	150	Down
*Rnaset2a*	17	0.025118864	-2.85	8,347,603	8,353,740	241	Down
*Prc1*	7	0.010471285	-2.82	79,959,186	79,960,588	150	Down
*St8sia4*	1	2.95E-05	-2.41	95,555,283	95,555,433	150	Down
*Lrrfip1*	1	0.00074131	-2.37	91,043,657	91,043,858	201	Down
*Acan*	7	0.004677351	-2.27	78,749,026	78,749,227	201	Down
*Slc25a27*	17	0.028183829	-2.13	43,956,575	43,956,725	150	Down
*Ppl*	16	0.039810717	-2.12	4,907,292	4,907,442	150	Down

### GO and KEGG pathway analyses of genes with different methylation

**Fig. 5:**
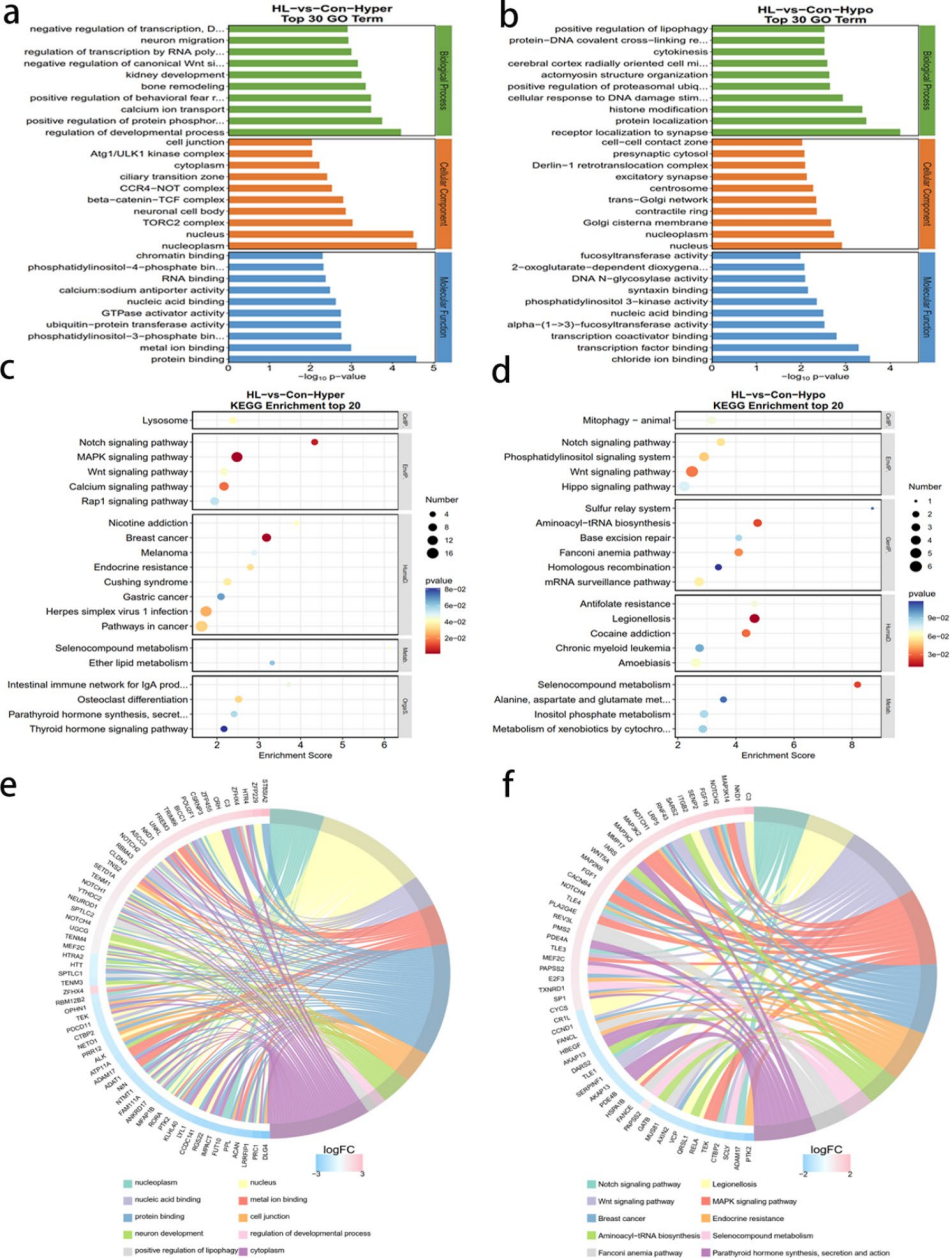
Biological information related to mRNA m6A methylation identified via GO and KEGG analyses. **(a)** GO analyses of genes with peaks related to m6A hypermethylation. **(b)** GO analyses of genes with peaks indicating m6A hypomethylation. **(c)** KEGG analyses of peaks related to m6A hypermethylation. **(d)** KEGG analyses of peaks reflecting m6A hypomethylation. **(e)** GO analyses chord diagram of genes with differentially methylated m6A peaks. **(f)** KEGG analyses chord diagram of genes with differentially methylated m6A peaks. BP, biological process; MF, molecular function; CC, cellular component; GO, Gene Ontology; KEGG, Kyoto Encyclopedia of Genes and Genomes

GO and KEGG pathway analyses of genes with different m6A methylation peaks were performed for determining the potential role of m6A modifications in the hippocampus after HL. The primary GO terms and KEGG pathways associated with hypermethylation are demonstrated in Fig. 5a and c, respectively. The primary GO terms and KEGG pathways associated with hypomethylation were demonstrated in Fig. 5b and d, respectively. GO analysis revealed peaks related to hypermethylation in HL mice that were significantly associated with regulating developmental processes, chromatin binding, and protein binding (Fig. 5a). Conversely, hypomethylation-associated peaks correlated significantly with receptor localization at synapses, fucosyl transferase activity, and chloride ion binding (Fig. 5b). KEGG analysis indicated that the hypermethylation-associated peaks in HL mice correlated significantly with the Notch-signaling and MAPK-signaling pathways (Fig. 5c). The hypomethylation-associated peaks correlated significantly with Legionellosis and the Wnt-signaling pathway (Fig. 5d). In summary, the GO analyses chord diagram demonstrated that the differentially methylated mRNAs between the HL and Con groups correlated with protein binding and regulating developmental progress (Fig. 5e). Additionally, KEGG analysis chord diagram demonstrated that mRNAs that exhibited altered m6A methylation were enriched in MAPK-signaling pathway (Fig. 5f).

### Altered mRNAs in mouse hippocampi after HL

**Fig. 6:**
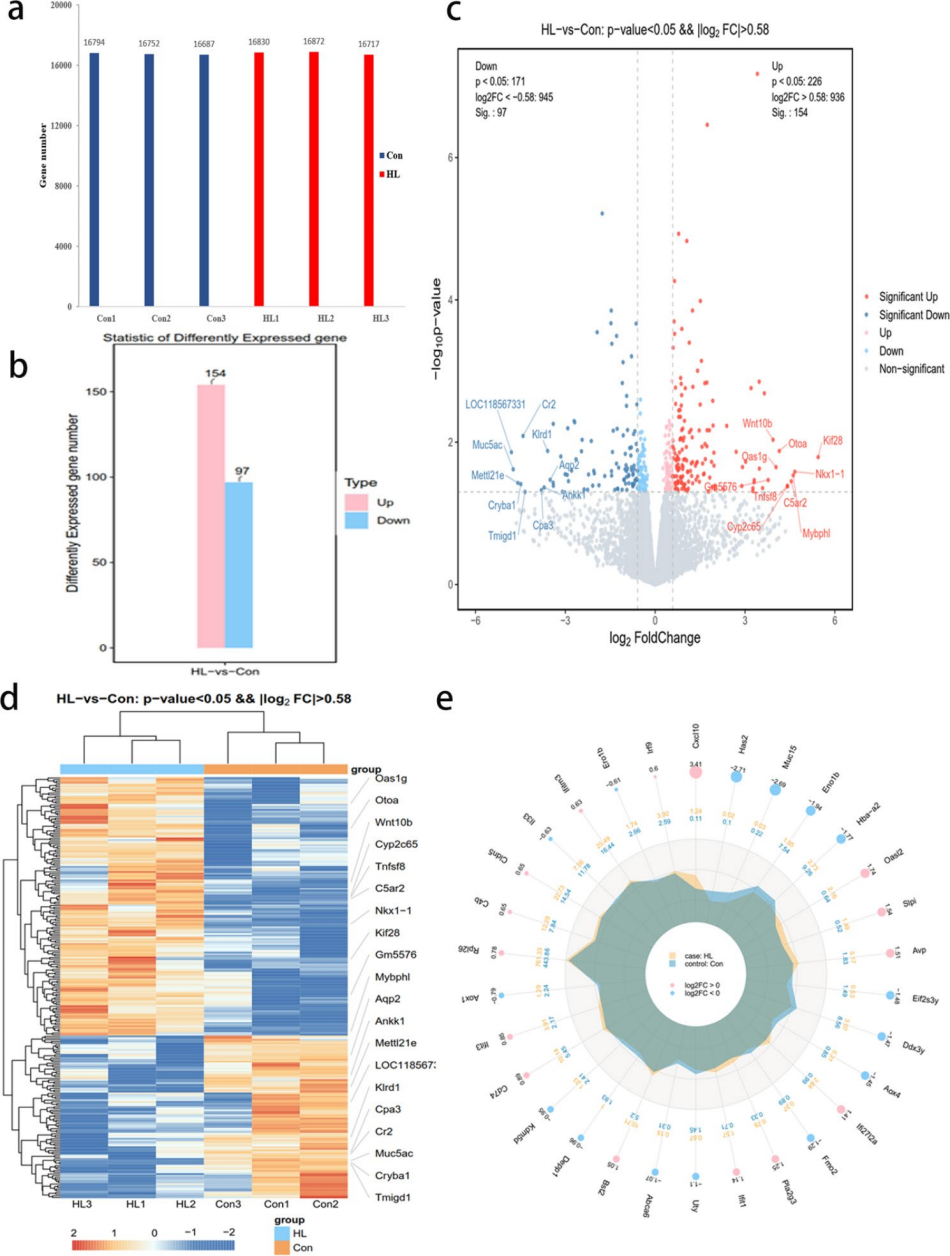
mRNA expression levels in mouse hippocampi after HL. **(a)** The number of genes with detectable mRNA levels in each sample in both groups. We detected the expression of an average of 16,744 and 16,806 genes in the Con and HL group, respectively. **(b)** The number of significantly different mRNA expression levels after HL, including 154 upregulated and 97 downregulated mRNAs (P < 0.05, |log_2_ FC| > 0.58) in mouse hippocampi after HL. **(c)** A volcano plot displaying significantly upregulated and downregulated mRNAs after HL. Red, upregulated genes; green, downregulated genes. **(d)** A heatmap displaying relative mRNA expression levels in both groups. Similar mRNA expression levels were found among the samples in both groups. **(e)** A radar map showing the 30 genes with the most significantly different expression levels between groups (*P* < 0.05)

The RNA-seq data from the input experiments were used to acquire transcriptome profiles (Additional file 4). On average, 16,806 and 16,744 mRNA transcripts were detected in the HL and Con mice, respectively (Fig. 6a). We identified 251 differentially expressed mRNAs in the HL group with 154 and 97 genes being upregulated and downregulated, respectively (*P* < 0.05, |log_2_ FC| > 0.58; Fig. 6b). Table 2 lists the top 20 most differentially expressed mRNAs. The volcano chart displays all significantly downregulated and upregulated mRNAs in the HL mice (Fig. 6c). We identified the top 10 most significantly upregulated (*Kif28*, *Nkx1-1*, *Mybphl*, *C5ar2*, *Cyp2c65*, *Tnfsf8*, *Otoa*, *Oas1g*, *Wnt10b*, and *Gm5576*) and downregulated genes (*LOC118567331*, *Muc5ac*, *Mettl21e*, *Cryba1*, *Cr2*, *Tmigd1*, *Cpa3*, *Ankk1*, *Klrd1*, and *Aqp2*). The heatmap shows the relative mRNA expression levels were comparable among samples in either group (Fig. 6d). The radar map displays the top 30 most differentially expressed genes between both groups (*P* < 0.05; Fig. 6e).

**Table 2:** The representative changed gene. (Top 20)

Gene_id	Chromsome	FoldChange	Log2FoldChange	P value	Regulation
*Kif28*	1	43.45455655	5.441435558	0.0162293	Up
*Nkx1-1*	5	25.33432945	4.663021739	0.0260227	Up
*Mybphl*	3	24.4256914	4.610327495	0.0291509	Up
*C5ar2*	7	23.35309053	4.545541583	0.0353948	Up
*Cyp2c65*	19	21.39526886	4.419219904	0.0416854	Up
*Tnfsf8*	4	21.29017576	4.412115955	0.0411565	Up
*Otoa*	7	17.6677598	4.143047218	0.0132915	Up
*Oas1g*	5	16.38921182	4.03467457	0.0224271	Up
*Wnt10b*	15	15.29475475	3.934965068	0.0092452	Up
*Gm5576*	6	13.67752784	3.773735587	0.0339823	Up
*LOC118567331*	1	0.035875729	-4.800848029	0.0138397	Down
*Muc5ac*	7	0.037414001	-4.740277948	0.0240738	Down
*Mettl21e*	1	0.041680633	-4.584479016	0.0373792	Down
*Cryba1*	11	0.044582308	-4.487384889	0.0387728	Down
*Cr2*	1	0.046931171	-4.413309729	0.0081753	Down
*Tmigd1*	11	0.049098081	-4.348189557	0.0498687	Down
*Cpa3*	3	0.07175006	-3.800876158	0.0472127	Down
*Ankk1*	9	0.076489594	-3.708592703	0.0433636	Down
*Klrd1*	6	0.082967284	-3.591313635	0.0132689	Down
*Aqp2*	15	0.087996741	-3.506406101	0.0337882	Down

### Combined analysis of the RNA-seq and MeRIP-seq

**Fig. 7:**
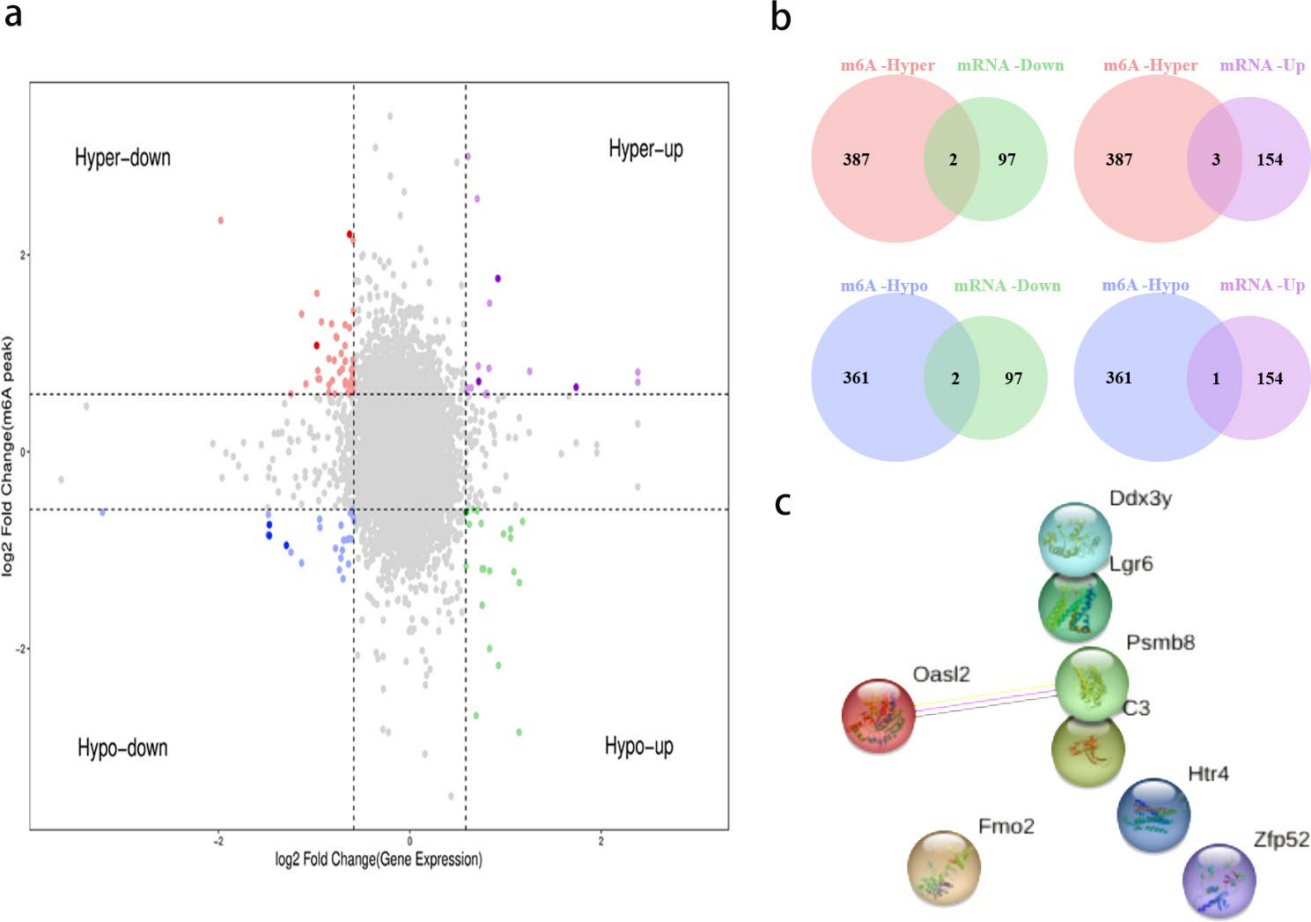
Combined MeRIP-seq and RNA-seq analyses of hippocampal samples after HL. **a, b)** The relationships between m6A-methylated mRNAs and their expression levels are displayed in a four-quadrant graph (**a**) and in a Venn diagram (**b**). **c)** Connections between differentially m6A-methylated mRNAs and the encoded proteins were analyzed by constructing a PPI network

RNA-seq (*P* < 0.05, |log_2_ FC| > 0.58) and MeRIP-seq data (*P* < 0.05, |log_2_ FC| > 0.58) were analyzed together. We identified eight genes with significantly altered m6A peaks and mRNA expression. Among them, three genes showed upregulated mRNA expression and m6A peaks, two showed downregulated mRNA expression and m6A peaks, another two showed upregulated mRNA expression with downregulated m6A peaks, and one showed downregulated mRNA expression with upregulated m6A peaks (Additional file 5). The quadrant graph and Venn diagram demonstrated relative changed genes (Fig. 7a, b). Based on these results, we speculate that these eight genes may be relative to the development of cognitive impairment after HL. A network of protein–protein interactions (PPI) was employed for the purpose of illustrating the relationship between the proteins that these eight genes encode (Fig. 7c).

### Expression of m6A methylation regulators altered in mouse hippocampi after HL

**Fig. 8:**
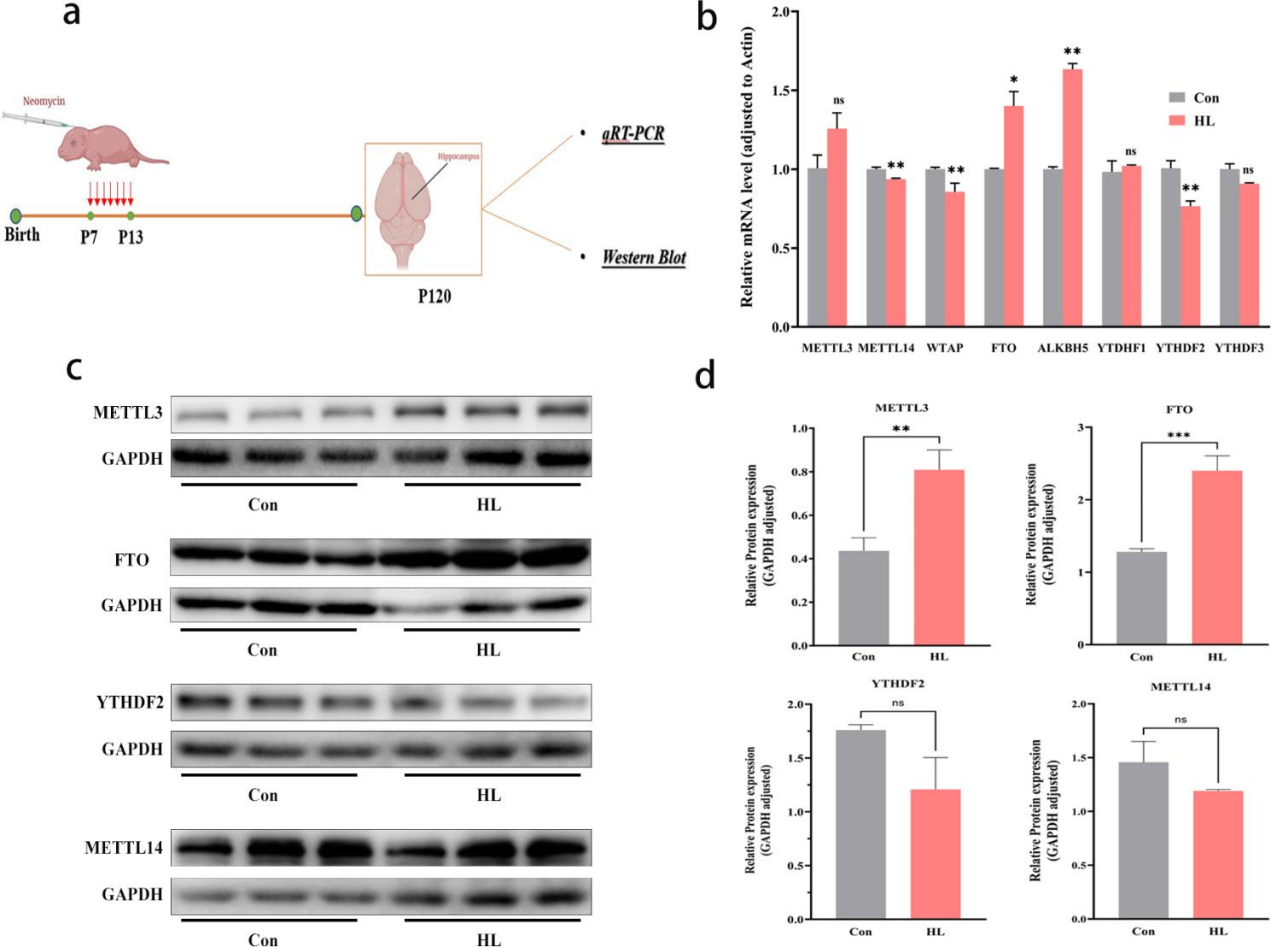
Regulated expression levels of m6A-methylated mRNAs in mouse hippocampi after HL. **(a)** Time points for qRT-PCR and western blot analyses after inducing HL. **(b)** qRT-PCR analysis showed that the hippocampal mRNA expression levels of *METTL14*, *WTAP*, and *YTHDF2* were significantly lower in the HL group than in the Con group and those of *FTO* and *ALKBH*5 were significantly higher. **c, d)** Western blotting results showed that the hippocampal protein expression levels of FTO and METTL3 were higher in the HL group than in the Con group, whereas those of METTL14 and YTHDF2 were comparable between both groups. The data shown were analyzed using an unpaired t-test and are presented as the mean ± SEM. Not significant (ns), *P* > 0.05; *P < 0.05; **P < 0.01; ***P < 0.001. n = 6 mice per group. Con, control; HL, hearing loss

We examined the levels of key m6A regulators (METTL14, METTLL3, WTAP, FTO, ALKBH5, YTDHF1, YTDHF2, and YTDHF3) in mouse hippocampi (Fig. 8a). Reverse transcription quantitative real-time polymerase chain reaction (qRT-PCR) analysis showed that *METTL14*, *WTAP*, and *YTHDF2* levels were significantly lower (*P* < 0.05); *FTO* and *ALKBH5* levels were significantly higher (*P* < 0.05); and *METTL3*, *YTDHF1*, and *YTHDF3* levels were not significantly different in HL mice than in control mice (*P*<0.05; Fig. 8b). Furthermore, the protein expression levels of METTL3, METTL14, FTO, and YTHDF2 in the hippocampus was measured via western blot. In HL group, METTL3 and FTO protein levels were significantly upregulated (*P* < 0.05), whereas METTL14 and YTHDF2 levels were downregulated (*P*<0.05), but not significantly (Fig. 8c, d). These results indicate that abnormal m6A methylation may be due to changes in METTL3 and FTO expression.

## Discussion

As far as we know, this research is the first to reveal the m6A epitranscriptome profiles of the mouse hippocampus following HL using MeRIP-seq analysis. We observed 748 altered m6A peaks including 387 peaks showing hypermethylation and 361 peaks showing hypomethylation. The differentially methylated mRNA molecules were functionally predicted using GO and KEGG pathway analyses.

Neomycin-induced HL in mice is a commonly used model of sensorineural HL [24]. In this experiment, the mice that received neomycin from P7 to P13 exhibited severe HL at P30 and P120 (Fig. 1). In a previous study, neonatal mice that experienced noise exposure could suffered from severe HL with decreased neurogenesis, chronic spatial learning, and memory deficits in adulthood [25]. *W*e used the PAT, a widely used behavioral test to detect learning and memory function in animals [26] to examine the cognitive function of mice at P120. Mice in the HL group showed a shorter latency period in the PAT test than the mice in the Con group (Fig. 2), suggesting that learning and memory deficits occurred after HL. Theories involving sensory deprivation, information degradation, common pathologies, and social isolation [27].

m6A methylation has been considered as novel epigenetic modifications related with various diseases, like cancers, inflammation, and cerebral ischemic disease [28–30]. With significant progress in sequencing technology, it has been identified that m6A is highly abundant in the mammalian brain [31]. The m6A is an abundant RNA modification in the brain, participating in neurodegenerative diseases. In mice with Huntington’s disease (HD), the alterations of m6A RNA methylation in hippocampal affected the expression levels of specific genes involved in synaptic plasticity and memory consolidation. Changes in expression and function of m6A RNA methyltransferase METTL3 and the m6A reader protein YTHDF1 involved were associated with memory deficits in HD mice [21]. In AD patients, the expression level of m6A RNA demethylase FTO decreased. For m6A RNA binding protein, the expression levels of YTHDC1, YTHDF1, FMR1, IGF2BP2, and HNRNPA2B1 rose, while YTHDF2 and EIF3H expression dropped [32]. In 3xTg AD mice, FTO has been shown to promote insulin-deficiency-associated AD by decreasing TSC complex subunit 1 (TSC1) mRNA levels, activating the mammalian target of the rapamycin (mTOR) signaling pathway, and promoting tau protein phosphorylation [15].

In this research, we examined m6A methylation profiles that may be involved in the mechanism of cognitive impairment after HL. According to the MeRIP-seq analysis, we revealed epitranscriptomic m6A dysregulation in the hippocampus of HL mice. By analyzing the m6A peak distribution profiles of mRNAs, we found that they were typically distributed in the CDS, 5′-UTR, and 3′-UTR region. Furthermore, the main variations in the m6A locations between the control and HL group were concentrated in the 3′-UTR (42.25%) (Fig. 4), which implied the potential target of m6A modifications in hippocampus after HL. Several cis elements responsible for posttranscriptional gene regulation are located in 3′ UTR [33]. As a critical region for RNA regulation, the 3’ UTR, plays a significant role in influencing translation efficiency, RNA stability, subcellular localization, and translation regulation [34]. Additionally, the 3’ UTR contains target sites for many RNA-binding proteins, which are key regulators of these important processes, which can bind to specific structural motifs or consensus sequences within the 3’ UTR [31]. Our results indicate m6A RNA methylation in the hippocampi may account for cognitive impairment in HL mice.

According to the result of GO analysis, those differentially methylated genes were primarily associated with synaptic and protein binding (Fig. 5). Synapse loss can induce cognitive impairment in many pathological processes such as AD [35], and impaired hippocampal synapses have been observed in patients with dementia [36]. According to the result of KEGG pathway analyses, those key differentially methylated genes were predominantly related to MAPK, Notch, and calcium-signaling pathways (Fig. 5). As a prominent intracellular signaling pathway, the MAPK pathway is of great importance in several critical physiological processes, including learning, memory, development, and cell differentiation [37, 38]. The MAPK cascade is required for spatial and contextual learning in mice and is associated with hippocampal neurodegeneration [39, 40]. It is noteworthy that Notch signaling pathways exhibited a correlation with both hyper- and hypomethylation of distinct genes (Fig. 5c, d), indicating a diverse role of m6A in the regulation of specific molecular processes. The Notch-signaling pathway participates in many biological processes, and deficiency in the pathway is detrimental to hippocampal neural-stem cell maintenance [41]. In addition, the key AD gene Notch 2, a genetic biomarker, was closely related to m6A regulators and might be the important targets in m6A methylation during the progression of AD [32]. In this study, the MeRIP-Seq analysis showed that there was hypermethylated peak of Notch2 in the region of the exons on chromosome 3 (Table S3). This genetic dynamic may impair cognitive function of the hearing loss patient and Notch2 may serve as a genetic marker for diagnosis.

The instability of mRNA can be determined by m6A modifications. To obtain a more comprehensive understanding of m6A methylation in the hippocampus after HL, conjoint analyses of MeRIP-seq and RNA-seq were utilized to screen all differentially m6A-methylated with differentially expressed mRNA. We had identified eight mRNAs that had undergone significant alterations in distribution of m6A peak and mRNA expression levels. However, we did not find significant correlations between mRNA expression and m6A methylation in the PPI network (Fig. 7c). The protein expression levels of those differentially m6A-methylated mRNAs should be determined by further researches.

m6A modification is a dynamic and reversible process driven via “writers,” removed via “erasers,” and recognized via “readers” [42]. Thus, we screened for several m6A regulators in Con and HL groups via performing qRT-PCR and western blot analyses. Among those m6A regulators, FTO is highly abundant in brain tissues and is related to neurotransmitter delivery and nervous system development [18, 43]. FTO dysfunction has been reported for participating in several mRNA processes including splicing, stability, translocation, and in protein translation. Several genome-wide association studies showed the importance of FTO in memory processing and its overexpression can significantly upregulate neuronal protein phosphorylation levels in mice with AD [15]. Exercise can decrease m6A levels by upregulating FTO in the mouse prefrontal cortex, and previous data showed that FTO knockdown in the prefrontal cortex led to enhanced consolidation of cued fear memory [14]. In this research, Western blot and qRT-PCR showed that the expression of FTO in the HL group increased compared to that in the control group. It is speculated that abnormal m6A modification in the hippocampus is due to the altered FTO expression after HL.

The current study has some limitations. First, relatively few mice hippocampal samples were used for MeRIP-seq analysis (three HL and Con samples each). Hence, the mRNAs showing altered m6A modifications should be further validated by performing MeRIP-PCR analysis with more samples. Second, we focused on some of the common key regulators of m6A and tested them by performing western blotting and qRT-PCR analyses. However, further in vitro and in vivo research are necessary to examine the specific roles behind m6A modification regulators (including “writers,” “erasers,” and “readers”) in greater detail to explore the mechanism underlying cognitive impairment after HL.

## Conclusions

In summary, this is the first study to present the m6A transcriptome-wide map about the mouse hippocampus following HL. The results provide novel insights into the dynamics of m6A modifications after HL, with indication that the FTO might play a critical role. Those findings expand our understanding of the role about m6A modifications and the underlying mechanisms contributing to cognitive impairment following HL from an epigenetic perspective. Further studies on the involvement of abnormal m6A mRNA methylation during the pathological process of hippocampal neurodegeneration following HL are warranted.

### Methods

#### Animals

Male, P7 Kunming mice were acquired from Vital River Laboratory Animal Technology Co., Ltd. (Beijing, China). Those mice were housed at the Animal Experimental Center of Fudan University in a temperature-controlled room (22–23 °C) with a 12-hour light/dark cycle. Water and food were both provided *ad libitum*. The Animal Care and Use Committee of Fudan University approved all procedures. The corresponding protocols were conducted to comply with the National Institutes of Health Guide for the Care and Use of Laboratory Animals to minimize potential animal suffering. The mice were randomly sorted into Con and HL groups for ABR testing (9 mice per group), PAT analysis (9 mice per group), MeRIP-seq and RNA-seq (9 mice per group), qRT-PCR analysis (3 mice per group), and western blot analysis (6 mice per group).

### Mouse model of neomycin-induced severe HL

The HL mouse model was established as previously described [24]. On P7, the experimental mice began receiving one daily dose of neomycin (Sangon Biotech, Shanghai, China) for seven consecutive days (200 mg/kg, subcutaneous, P7–P13). A corresponding dose of sterile saline was administered to the control group mice. ABR tests were performed as previously described [44] on P30 and P120 after anesthetizing the mice with xylazine (25 mg/kg) and esketamine (50 mg/kg). The average response to 1,000 repetitive stimuli at each frequency were magnified (100,000 times), filtered (0.3–3.0 kHz), and then digitized with an analog-to-digital converter (Tucker-Davis Technologies, Alachua, FL, USA). Specific evoked brainstem responses were calculated at frequencies of 8, 16, 24, and 32 kHz. At each frequency, the stimulus intensity was initiated from 90 dB and increased at 5 dB intervals until 20 dB.

### Cognitive function assessment with the PAT

Cognitive function was determined using the PAT [45, 46] at P120. During the training period, mice were arranged in the left light box. The door opened automatically after an adaptation period (30 s), and the system recorded the latency before each mouse crossed into the dark zone. Once the mice entered the right dark zone, automatic door would be closed immediately, and the electrical shock (0.7 mA, 2 s) was released. Electrical shock stimuli were not applied during the test period. The maximum latency did not exceed 300 s.

### Western blot analysis

The mice were sacrificed at P120 under *isoflurane* anesthesia, and the bilateral hippocampi were quickly obtained. Western blotting was conducted as previously described [44]. Radioimmunoprecipitation Assay lysis buffer (Beyotime, Shanghai, China) containing phenylmethylsulfonyl fluoride was used to extract the total protein. Primary antibodies against METTL14 (1:1000 dilution; ABclonal, Wuhan, China; A8530), METTL3 (1:1000 dilution; ABclonal; A19079), FTO (1:500 dilution; Proteintech, Wuhan, China; 27226-1-AP), YTHDF2 (1:4000 dilution; Proteintech; 24744-1-AP), and GAPDH (1:5000 dilution; Proteintech; HRP-60,004) were used. The horseradish peroxidase-conjugated goat anti-rabbit IgG (1:10000 dilution; Jackson, Pennsylvania, USA; 111-035-003) were used as the secondary antibody. The expression levels of YTHDF2, FTO, METTL14, and METTL3 were normalized to those of GAPDH to control for loading the difference amount of total protein in the gels.

### qRT-PCR analysis

The mouse hippocampi were harvested at P120. Total RNA of hippocampi was obtained via using the TRIzol method (Invitrogen, Waltham, MA, USA) and detected by NanoDrop 2000 instrument (Thermo Fisher Scientific, Waltham, MA, USA). In reverse transcription procession, a PrimeScript™ RT Reagent Kit With gDNA Eraser (Perfect Real Time, Takara Bio, Shiga, Japan; RR047A) was applied to synthesize the complementary DNA (cDNA). TB Green Master Mix (Tli RNase H Plus, Takara Bio, Shiga, Japan; RR420A) in an ABI 7500 Real-Time PCR system (Applied Biosystems, Foster City, CA, USA) was used for qRT-PCR analysis. As a normalization control, β-actin was chosen and the relative genes expression were calculated by the 2^−ΔΔCT^ method. The primer sequences are listed in Additional file 6.

### MeRIP-seq and RNA-Seq with data processing

#### MeRIP-seq and RNA-Seq

The hippocampal samples were harvested at P120. Each sample was collected from the hippocampi of three mice each in the Con and HL group. The number of samples in each group was as follows: Con, n = 3; HL, n = 3”. Total RNA was extracted and isolated using magnetic beads with poly-T oligonucleotides and then chemically fragmented into 150-nucleotide oligonucleotides. RNA quantity and quality were analysed by an Agilent 2100 Bioanalyzer (Agilent Technology, Santa Clara, California, USA) and a NanoDrop 2000 instrument (Thermo Fisher Scientific, Waltham, MA, USA). The MeRIP-seq and RNA-seq analyses were conducted by OE Biotech Co., Ltd. (Shanghai, China).

For MeRIP-seq, the cleaved RNA fragments were incubated for 2 h at 4 °C with an m6A-specific antibody (Synaptic Systems, Germany; 202,003) in the immunoprecipitation (IP) buffer. The mixture was then incubated with protein A beads and eluted with elution buffer (1× IP buffer containing 6.7 mM m6A). Eluted m6A-containing fragments (IP fraction) and untreated input control fragments were converted to final strand-specific cDNA libraries in the presence of dUTP. The average insert size of the paired-end libraries was approximately 150 base pairs. We then performed paired-end 2 × 150 bp sequencing using an Illumina NovaSeq 6000 platform in accordance with the manufacturer’s recommended protocol.

For RNA-seq, the input sample without IP were used for sequencing library generation with the NEBNext® Ultra II Directional RNA Library Prep Kit (New England Biolabs, Inc., USA).

### Bioinformatic analysis

Raw reads obtained from RNA sequences were used to statistical and quality control analyses. Adaptor, low quality bases and poly-N sequences were trimmed using FastP [47] to obtain clean data. After further removing rRNA reads, HISAT2 was applied to make clean reads map to reference genome [48], and only high-mapping unique reads were retained. To access the quality of MeRIP-seq, the deepTools [49] and Guitar R package [50] were used. In each m6A-IP sample, the enriched m6A peaks were identified via MeTDiff peak-calling software [51]. The threshold criteria for calling peaks showing differential m6A methylation were an absolute log_2_ FC value of > 0.58 and *P* value of < 0.05. Peaks showing differential methylation between groups were annotated using ChIPseeker software.

Differential gene expression was analyzed by using DESeq2. *P* < 0.05 and |log2 FC| > 0.58 was considered as the threshold for significant differentially expressed genes (DEGs). Hierarchical cluster analysis of DEGs was performed using R (v 3.2.0) to demonstrate the gene expression pattern in different groups and samples. The radar map of top 30 genes was drawn to show the expression of upregulated or downregulated DEGs using R packet ggradar.

### Functional enrichment analysis

The identified and differential peaks with the hypergeometric distribution were analyzed by R software by performing GO and KEGG pathway analyses [52–54]. Both MEME and DREME software were applied to observe sequence motifs which were annotated via Tomtom software.

#### Combined analysis of MeRIP-seq and RNA-seq data

Those mRNAs with different expression levels were categorized as upregulated or downregulated based on the RNA-seq data. Differential regulation of gene methylation was identified based on changes found in m6A peak abundances following MeRIP-seq. The Python Script (v.2.7.12) software was used to perform correlation analysis on data obtained from both sequencing approaches to compare transcription and methylation levels simultaneously. To construct a PPI network, we utilized the Search Tool for the Retrieval of Interacting Genes database (https://www.string-db.org).

### Statistical analysis

The data generated in this experiment were analyzed with GraphPad Prism® 9 (San Diego, CA, USA) and were displayed as the mean ± standard error of the mean (SEM). The unpaired Student’s t test was employed to assess differences between the two groups. A *P* value < 0.05 was used to representative a statistically significant difference.

### Electronic supplementary material

Below is the link to the electronic supplementary material.

Supplementary Material 1

Supplementary Material 2

Supplementary Material 3

Supplementary Material 4

Supplementary Material 5

Supplementary Material 6

Supplementary Material 7

## Data Availability

The raw sequence data reported in this paper have been deposited in the Genome Sequence Archive in BIG Data Center (https://ngdc.cncb.ac.cn/gsa/s/2Mz4btx4) under the accession number: CRA011232.

## List of abbreviations

ABR: auditory brainstem response
ALKBH5: α-ketoglutarate-dependent dioxygenase alkB homolog 5
BP: biological process
CC: cellular component
cDNA: complementary DNA
CDS: coding sequence
Con: control
Diffpeak: peak reflecting differential m6A methylation
FC: fold-change
FTO: fat-mass and obesity-associated protein
GO: Gene Ontology
HL: hearing loss
KEGG: Kyoto Encyclopedia of Genes and Genomes
MeRIP-seq: methylated RNA immunoprecipitation sequencing
METTL14: methyltransferase-like 14
METTL3r: methyltransferase-like 3
MF: molecular function
ns: not significant
P: postnatal day
PAT: passive-avoidance test
RNA-seq: RNA sequencing
PPI: protein–protein interaction
qRT-PCR: reverse transcription quantitative real-time polymerase chain reaction
SEM: standard error of the mean
SPL: sound pressure level
UTR: untranslated region
WTAP: Wilms tumor 1-associating protein
YTHDF: YTH domain family
