# Supplementary material for: Comprehensive analysis of N6-methyladenosine-related RNA methylation in the mouse hippocampus after acquired hearing loss
Source: BMC Genomics. 2023 Sep 27;24:577. doi: 10.1186/s12864-023-09697-4 (PMC10537436; doi:10.1186/s12864-023-09697-4)
Supplement: Supplementary file 6 — Supplementary Material 6 [file 12864_2023_9697_MOESM6_ESM.docx]

**Table S6** The primes for qRT-PCR

| **Genes** | **Primer types** | **Primer Sequences (5’-3’)** |
| --- | --- | --- |
| *METTL3* | Forward | CGCTGCCTCCGATGTTGATCTG |
| *METTL3* | Reverse | TCTCCTGACTGACCTTCTTGCTCTG |
| *METTL14* | Forward | ACACCCCGACCTGACTTGACTG |
| *METTL14* | Reverse | CCCACTCCTGCCACTCCTCTG |
| *WTAP* | Forward | AAAGTAACCTGGCTTCCTTCCCTTG |
| *WTAP* | Reverse | TGCCTGCCTCTGCCTCCTAAG |
| *FTO* | Forward | ACCACTGTCACCTCTTTCCTCCTC |
| *FTO* | Reverse | TCCTTGTAGCTCCTTCCTCTTCCTG |
| *ALKBH5* | Forward | TGTTCTTGGCTTTCCTCCTTGATGG |
| *ALKBH5* | Reverse | TGTCTCTACTGGCTACTCTGGTGTG |
| *YTDHF1* | Forward | CCCTGTCCTGGAGAAACTGAAAGC |
| *YTDHF1* | Reverse | GTACTTGATGGAGCGGTGGATGTC |
| *YTDHF2* | Forward | TTGCCTCCACCTCCACCACAG |
| *YTDHF2* | Reverse | CCCATTATGACCGAACCCACTGC |
| *YTDHF3* | Forward | GCAGTTACGGCTATCCACCTAGTTC |
| *YTDHF3* | Reverse | AGTCCAGTCATGCCTTGCTCAATAC |
| *β-actin* | Forward | CCTCTATGCCAACACAGT |
| *β-actin* | Reverse | AGCCACCAATCCACACAG |
