# Supplementary material for: Comprehensive analysis of N6-methyladenosine-related RNA methylation in the mouse hippocampus after acquired hearing loss
Source: BMC Genomics. 2023 Sep 27;24:577. doi: 10.1186/s12864-023-09697-4 (PMC10537436; doi:10.1186/s12864-023-09697-4)

**Supplemental figures. Representative immunoblots.**

In the Western blot experiment, we initially aimed to visualize all four target bands on a single membrane. Unfortunately, the attempts were unsuccessful. Consequently, we cut the gel based on the protein marker before proceeding with antibody incubation. Finally, four membranes were utilized to achieve the final image. The striped bands within the red rectangle refer to the gel images used in the manuscript.


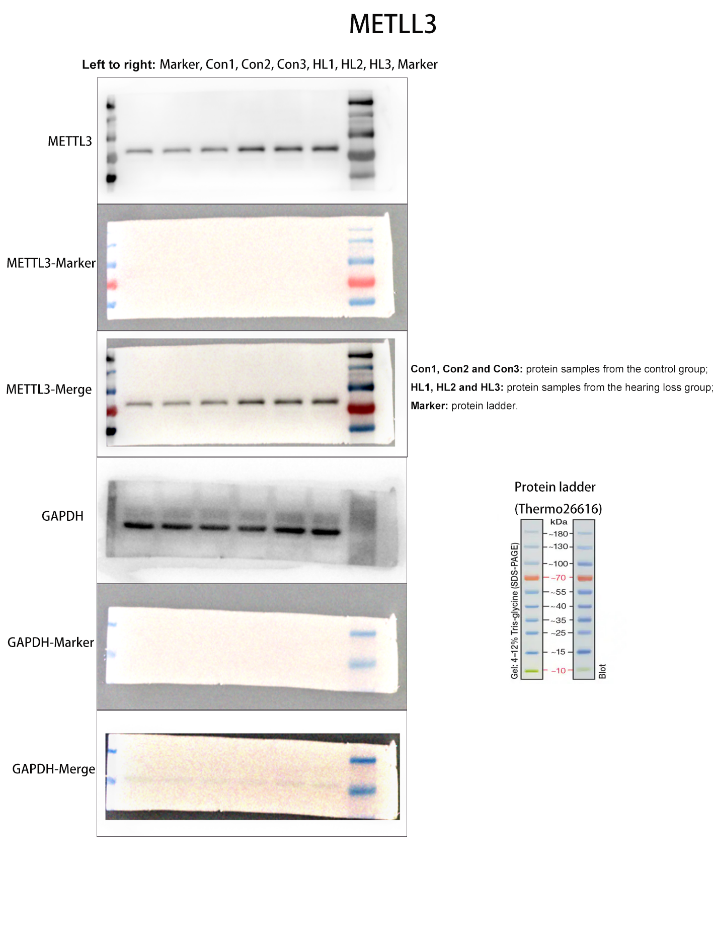


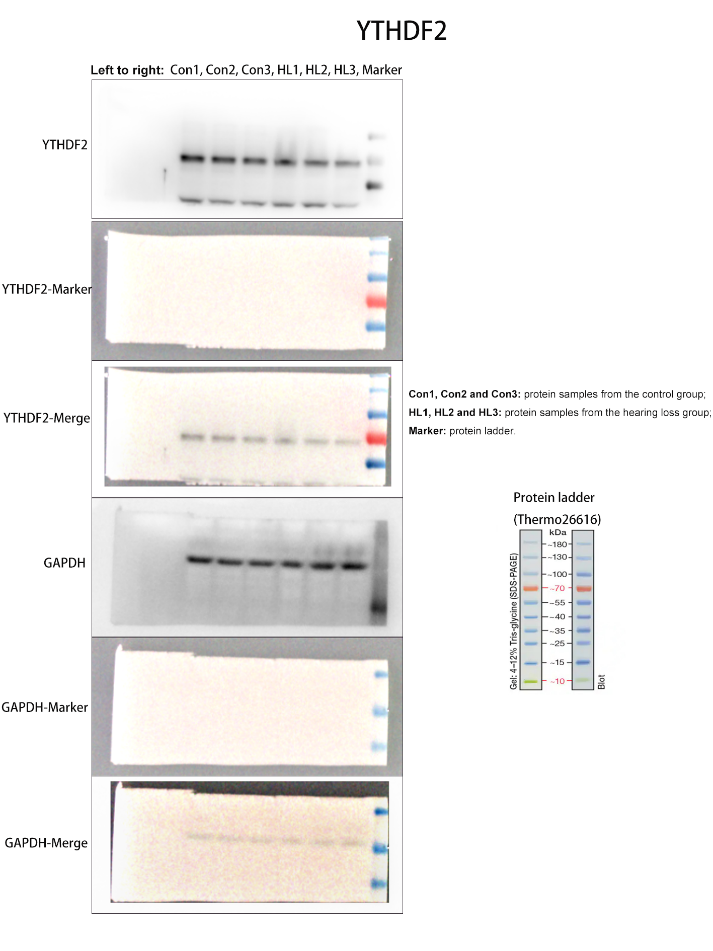


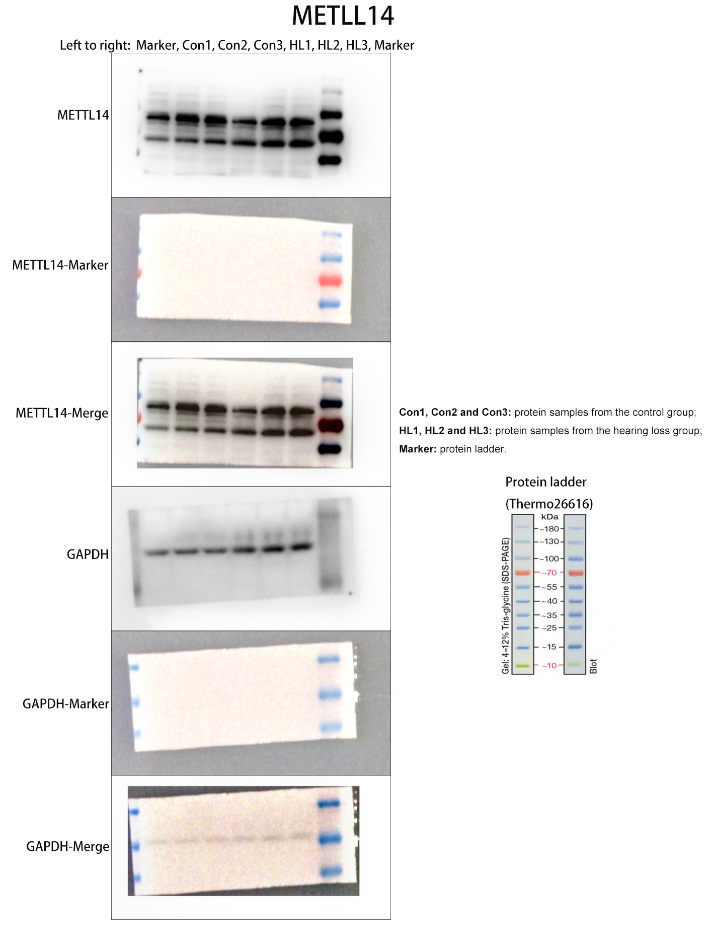


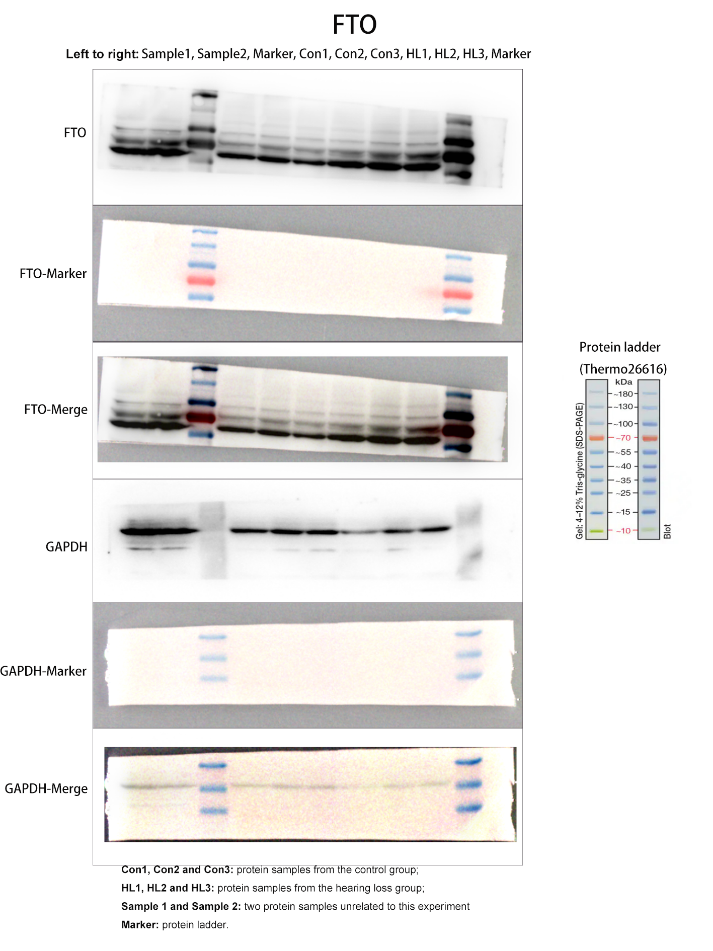

Supplement: Supplementary file 7 — Supplementary Material 7 [file 12864_2023_9697_MOESM7_ESM.docx]
